# Deciphering skin architecture, wound pathophysiology and molecular mechanisms of healing: insights into immune response and therapeutic strategies

**DOI:** 10.3389/fbioe.2026.1812108

**Published:** 2026-06-03

**Authors:** Mayank Maan, Shubhi Joshi, Avneet Saini

**Affiliations:** 1 Department of Biophysics, Panjab University, Chandigarh, India; 2 University School of Allied Health Sciences, Rayat Bahra University, Mohali, Punjab, India

**Keywords:** advanced wound dressings, chronic wounds, regenerative medicine, wound healing, wound therapeutics

## Abstract

Skin not only serves as a protective barrier, but also as a dynamic immunological and sensory organ responsible for complex physiological processes. Disruption through trauma, surgery, burns or chronic metabolic disease initiates a tightly regulated healing cascade involving inflammatory signaling, extracellular matrix remodeling, angiogenesis and coordinated cellular responses. While acute wounds generally progress through orderly phases of repair, chronic wounds such as diabetic foot ulcers, venous ulcers and pressure injuries become arrested in persistent inflammatory states characterized by protease imbalance, senescent cell accumulation, impaired vascularization and colonization of microbial biofilms, contributing to significant global morbidity and healthcare burden. This review integrates structural biology, immune mechanisms and molecular signaling pathways that govern wound repair, clearly delineating the differences between acute and chronic healing. Particular emphasis is placed on inflammation-driven dysregulation, extracellular matrix dynamics and defective angiogenesis that underpin chronic wound pathology. Rare and often underrepresented wound types, including radiation-induced and autoimmune-associated lesions, are also discussed to provide a broader and clinically relevant perspective. Importantly, the review highlights translational and technological advances transforming wound management, including self-healing hydrogels, nanofiber scaffolds, bioactive and stimuli-responsive dressings, biosensor-integrated platforms, cellular and molecular therapies and AI-assisted diagnostic systems. These innovations exemplify a shift from passive wound coverage toward biointeractive, precision-guided and microenvironment-responsive therapeutic strategies. By bridging immunopathology, regenerative medicine, nanobiotechnology and digital health platforms within a single cohesive narrative, this review serves as a comprehensive and integrative resource for academicians, clinicians, translational researchers and pharmaceutical innovators seeking a mechanistically grounded yet application-oriented understanding of modern wound care.

## Graphical Abstract

**Figure:**
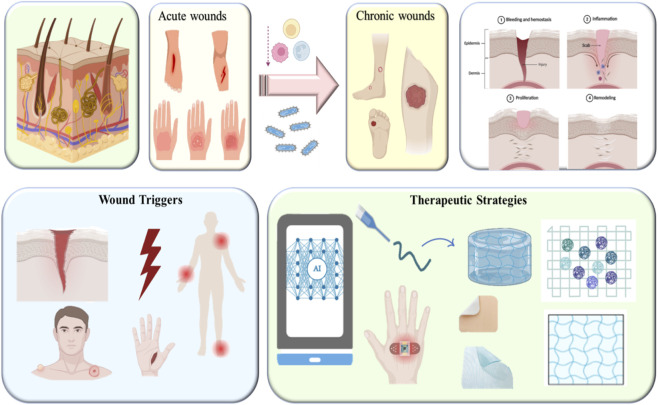

## Highlights

- This article reviews structural and immune roles of skin in orchestrating wound healing.
- It explains mechanisms distinguishing acute healing from chronic wound pathophysiology.
- It discusses rare wound types, including radiation-, autoimmune- and neoplastic-lesions.
- Further, it summarizes emerging therapeutic strategies such as bioengineered scaffolds, smart dressings and regenerative interventions.
- Finally, it discusses translational relevance showing how mechanistic insights and therapeutic innovation converge to advance personalized wound care.

## 1 Introduction

Human skin is a highly complex and multifunctional organ that serve as the first line of defence against external environment (McKnight et al., 2022). It makes up around 15% of the total body weight and roughly covers a surface area of approximately 1.5–2 square meters. Beyond acting as a physical barrier, the skin plays vital roles in regulating body temperature, sensing environmental stimuli, supporting immune defence, maintaining fluid balance, producing vitamin D and contributing to hormonal signalling (McKnight et al., 2022; Peate, 2025). Structurally, skin is constructed of three main layers, the epidermis, dermis and hypodermis. Each layer contains specialized cell types, extracellular matrix (ECM) components and blood vessels that work together to maintain tissue structure and overall physiological stability. When the skin is injured by trauma, the body initiates a highly coordinated and time-dependent healing process (Peña and Martin, 2024). This involves four overlapping phases, namely, hemostasis, inflammation, proliferation and remodeling. Effective healing depends on the synchronized activity of various cells, including keratinocytes, fibroblasts, endothelial cells and immune cells, as well as the signalling molecules and ECM that support them. Disruption in any step leads to poor healing outcomes such as excessive scarring, abnormal tissue formation or formation of chronic wounds (Raziyeva et al., 2021). Acute wounds include surgical incisions, abrasions and superficial burns. They generally follow a predictable healing process that leads to functional and cosmetic recovery. On the contrary, chronic wounds like diabetic foot ulcers; pressure, arterial and venous leg ulcers fail to progress through the normal stages of repair. These wounds are marked by persistent inflammation, impaired blood vessel formation, microbial colonization and disorganized ECM remodeling (Raziyeva et al., 2021). As a result, they are difficult to treat and carry a high risk of complications such as infection or amputation and even mortality (Barman et al., 2023). Associated economic burden is considerable, with the annual cost of wound care in the United states being, estimated at approximately $50 billion in 2023 (Campagna et al., 2023). This burden is expected to grow with the rising prevalence of diabetes, vascular diseases and aging populations (White, 2024).

Despite expanding knowledge of wound pathophysiology, critical gaps remain. Emerging evidence highlights the roles of immune dysregulation, oxidative stress, mitochondrial dysfunction and microbiome imbalance in delayed healing (Malik et al., 2023; Jang et al., 2024). Chronic wounds often represent a failure to transition from pro-inflammatory to pro-resolving states. Advances in single-cell sequencing and spatial transcriptomics have further revealed pronounced cellular and molecular heterogeneity within wound microenvironments, underscoring the need for precision-guided interventions (He et al., 2024). In response, therapeutic innovation has accelerated. Advanced biomaterials such as smart hydrogels, nanofiber scaffolds and stimuli-responsive dressings are being engineered to actively modulate the wound niche (Barman et al., 2025). Cell-based approaches, including mesenchymal stem cell therapies and targeted immunomodulation, are being explored to restore regenerative competence (Abusalah et al., 2024; Banerjee et al., 2024). Concurrently, digital technologies incorporating artificial intelligence and machine learning are being integrated into wound assessment to enable predictive monitoring and personalized treatment. However, translation from laboratory success to clinical reliability remains challenging, with regulatory, manufacturing and population heterogeneity barriers limiting widespread adoption (Ding et al., 2022; Las Heras et al., 2024).

**FIGURE 1:**
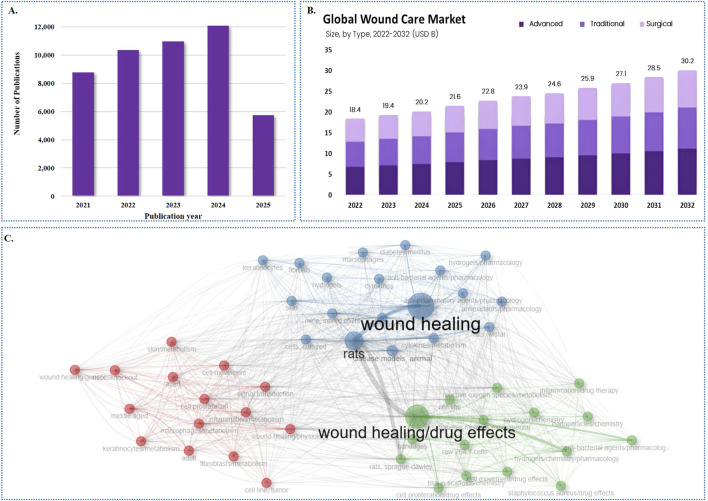
Publication trends related to skin and wound research (2021 -2025) based on Web of Science Core Collection **(A)**. Projected growth of the global wound care market by type (2022 -2032) **(B)**. Keyword co-occurrence network from literature on wound healing between 2021 and 2025, showing interconnected research themes related to inflammation, wound healing, animal models, biomaterials and therapeutic interventions **(C)**.

Recent bibliometric and market analyses underscore the expanding scientific and economic momentum in wound healing research. Publication trends retrieved from the Web of Science Core Collection demonstrate a steady increase in studies addressing skin biology, immunology and wound repair (Figure 1A). Parallel market projections indicate that the global wound care market is expected to exceed $30 billion by 2032, up from approximately $21 billion in 2022 (Figure 1B), reflecting strong translational and commercial interest. Keyword co-occurrence mapping further revealed interconnected research themes within contemporary wound healing literature (Figure 1C), with major clusters centered around inflammation, wound healing, animal models, biomaterials, hydrogels and antimicrobial therapies. The network demonstrates strong relationships between preclinical studies, clinical and physiological investigations, and material-based therapeutic strategies. Together, these analyses highlight the interdisciplinary breadth, translational relevance and sustained investment shaping the future of wound science.

In this review, we attempt to provide a comprehensive, multidisciplinary overview of skin architecture, wound pathophysiology and molecular repair mechanisms, including intrinsic and extrinsic factors that hinder the overall healing process. We further highlight the emerging strategies in biomaterial-based wound care, regenerative medicine, immunotherapy and smart dressings. This review was undertaken to address the growing need for an integrated understanding of the biological, immunological and therapeutic dimensions of wound healing, which are often studied in isolation but must work together in clinical reality. Through this study, we aim to bridge current biological knowledge with translational applications and comprehensively discuss the route towards personalized, regenerative wound care.

## 2 Skin physiology, molecular mechanisms and functions

### 2.1 Skin composition and architecture

**FIGURE 2:**
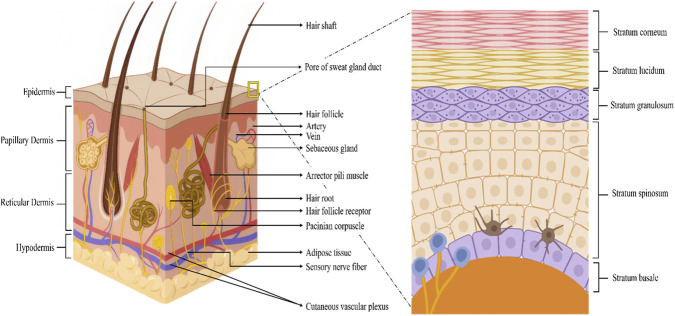
Pictorial representation of epidermis, consisting of stratified layers, with proliferating basal keratinocytes in the *stratum basale* migrating upwards through the *stratum spinosum* and *granulosum* to the *stratum corneum* (Joshi et al., 2025). The dermis, subdivided into papillary and reticular layers, contains vasculature and skin appendages such as hair follicles. The hypodermis comprises adipocytes and provides insulation and structural support. Reprinted with permission (Joshi et al., 2025).

The skin is the largest organ of the human body, covering approximately 1.5–2 m^2^ and accounting for nearly 15% of total body weight. Structurally, it is composed of three main layers: the epidermis, dermis and hypodermis (Figure 2) (McGrath and Uitto, 2023; Shah et al., 2024). Together, these layers provide mechanical protection, prevent excessive water loss and serve as the first interface between the body and external environment (Biggs et al., 2020). The epidermis and dermis are connected through the basement membrane zone, while the hypodermis forms the deeper subcutaneous layer that provides cushioning, insulation and metabolic support (Yazdi and Baqersad, 2022; Lotfollahi, 2024).

The epidermis is a stratified squamous epithelium that lacks blood vessels and relies on diffusion from the dermis for nutrient supply (Lim, 2021). Its thickness varies depending on anatomical location, being thinner on the eyelids and thicker on the palms and soles (Lai-Cheong and McGrath, 2021). Keratinocytes constitute nearly 95% of epidermal cells and undergo continuous renewal (Al-Dhubaibi et al., 2025). These cells originate in the basal layer and progressively differentiate as they migrate toward the surface, ultimately forming the outermost barrier (Yin et al., 2023). In addition to keratinocytes, the epidermis contains melanocytes, Langerhans cells and Merkel cells (Table 1).

**TABLE 1:** Different cell types present in epidermis and their functions.

Cell type	Presence	Function	References
Keratinocytes	Approximately 95% of epidermal cells are keratinocytes. They originate from stratum basale, which is deepest layer of epidermis.	- They begin in stratum basale as undifferentiated keratinocytes and start to differentiate into polyhedral-shaped keratinocytes as they migrate up to stratum spinosum. - In stratum granulosum, they contain granules with structural proteins like trichohyalin, making them flat. - Their major function is to act as a barrier, preventing the loss of water and the entry of contaminants. - They produce and store keratin and regulate calcium absorption for vitamin D synthesis.	Liarte et al. (2020), Yin et al. (2023), Al-Dhubaibi et al. (2025)
Langerhans’ cells	Originate from bone marrow, migrate to stratum basale and localize there. Also present sporadically in the stratum spinosum.	- Act as the first line of defense against invading microorganisms through their phagocytic ability. - Decrease in Langerhans cells with aging, thereby reducing skin’s immune function in elderly.	Neagu et al. (2022)
Melanocytes	Found in stratum basale, the deepest layer of epidermis.	- Contain melanin, which is the pigment responsible for skin, hair and eye color. It protects skin by absorbing UV light and minimizing free radical formation in the basal layer. - Reduction in number of melanocytes with aging, contributing to diminished immune function of skin.	Sevilla et al. (2022), Zhang et al. (2025)
Merkel cells	Found in small numbers in stratum basale in both hairless and hairy skin sections.	- Connected to surrounding keratinocytes by desmosomes and act as slowly adapting mechanoreceptors involved in sensation. - Studies suggest that they help attract nerve endings into the epidermis and stimulate keratinocyte growth.	Oss-Ronen and Cohen (2021), Bataille et al. (2022)

Morphologically, the epidermis is organized into distinct layers reflecting stages of keratinocyte differentiation. The outermost *stratum corneum* consists of flattened, enucleated corneocytes embedded in a lipid matrix, forming the principal barrier against water loss and microbial entry. Continuous turnover of this layer maintains epidermal homeostasis (Lefèvre-Utile et al., 2021; Quan, 2023). The *stratum basale* contains proliferative cells anchored to the basement membrane. Above it lies the *stratum spinosum*, where keratinocytes form strong intercellular junctions that maintain tissue cohesion. The stratum granulosum contains cells rich in keratohyalin and lamellar granules, which contribute to lipid barrier formation (Matsui, 2023; Lotfollahi, 2024). The basement membrane separates the epidermis from the dermis and provides structural anchorage and signals essential for tissue stability and repair (Kumar, 2024). Age-related thinning of this interface weakens epidermal attachment and increases skin fragility (Lotfollahi, 2024).

Beneath the epidermis lies the dermis, a connective tissue layer rich in ECM (Sullivan and Myers, 2022; Liu et al., 2024). Unlike epidermis, the dermis is vascularized and provides structural strength and elasticity. Collagen types I and III confer tensile resistance, while elastin fibres allow stretch and recoil. Proteoglycans and glycoproteins regulate hydration and matrix organization. (Hinz, 2021; Kumar, 2024).

Structurally, dermis is subdivided into the papillary and reticular regions (Figure 2) The papillary dermis contains loose connective tissue, capillaries and immune cells, supporting nutrient exchange and early wound responses (Sullivan and Myers, 2022). The reticular dermis is composed of dense collagen bundles and houses most of the skin appendages and vascular networks, providing mechanical resilience (McGrath and Uitto, 2023).

Fibroblasts are the principal cellular component of dermis and play a central role in synthesizing and remodeling of ECM (Boraldi et al., 2024). Increasing evidence indicates functional heterogeneity among fibroblast subpopulations, with distinct roles in matrix deposition, immune modulation and wound repair (Jiang and Rinkevich, 2021).

### 2.2 Extracellular matrix: composition, structure and functions at a glance

**FIGURE 3:**
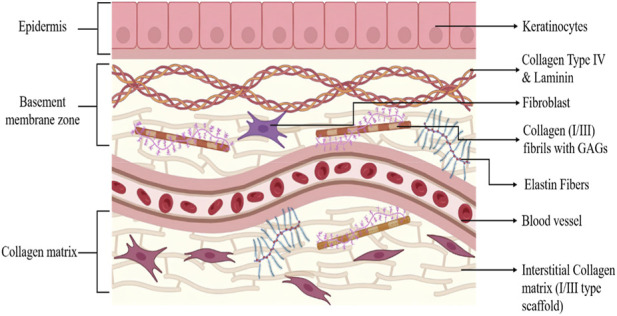
Schematic representation of the ECM within human skin. From top to bottom: the epidermal keratinocytes form the outermost protective barrier. Beneath them lies BMZ, composed primarily of collagen IV and laminin. In the underlying dermis, fibroblasts synthesize fibrillar collagens (types I and III), shown as pink fibres and proteoglycans such as decorin and biglycan, shown as purple side branches. Elastic fibers (blue mesh) contribute to tissue recoil and flexibility. Interspersed throughout the ECM is a blood vessel containing red blood cells, surrounded by a fibrous collagen network that supports tissue structure and immune cell migration.

The extracellular matrix (ECM) of skin is a highly specialized, hierarchically organized and dynamic meshwork that provides structural integrity while actively regulating cell behaviour, immune responses and tissue repair (Huang et al., 2022). It is broadly divided into two main compartments, basement membrane zone (BMZ), which separates the epidermis from the dermis and the interstitial dermal ECM (Pfisterer et al., 2021) (Figure 3).

The BMZ forms a specialized interface composed mainly of collagen IV, laminins, nidogens and perlecan. This sheet-like structure anchors basal keratinocytes to the underlying dermis and maintains epithelial polarity and stability (Yang et al., 2022). Beyond structural support, the BMZ provides signaling cues that regulate keratinocyte migration and differentiation (Sullivan and Myers, 2022). Re-establishment of this interface is a critical step during wound healing, as proper re-epithelialization depends on basement membrane.

Importantly, the ECM is not an inert scaffold. It functions as a reservoir for growth factors, cytokines and chemokines, regulating their spatial distribution and bioavailability (Potekaev et al., 2021). Through interactions with integrins and other surface receptors, the ECM influences keratinocyte proliferation, fibroblast activation and endothelial cell behavior (Hegde et al., 2021). It also directly modulates immune cell trafficking and polarization, shaping the inflammatory microenvironment. During normal wound repair, ECM remodeling is tightly controlled by matrix metalloproteinases (MMPs) and their inhibitors. This balance allows removal of damaged matrix components and deposition of new collagen. In chronic wounds however, excessive protease activity leads to uncontrolled matrix degradation, impaired angiogenesis and persistent inflammation. The resulting microenvironment is mechanically unstable and biologically hostile, preventing effective tissue regeneration. Emerging imaging and single-cell approaches have revealed that the ECM is spatially heterogeneous and forms distinct microenvironments within the skin. These localized niches influence stromal and immune cell function and may determine healing outcomes (Potekaev et al., 2021).

### 2.3 Skin aging: structural, molecular and immune dysregulation

Skin aging is a complex, multifactorial process driven by intrinsic factors such as genetics, hormonal changes or cellular senescence and extrinsic stressors including ultraviolet (UV) radiation, air pollution and oxidative stress (Maan et al., 2024). These combined influences lead to visible changes such as wrinkling, thinning, dryness and loss of elasticity, supported by structural and molecular degeneration (Hussein et al., 2025). At the structural level, aged skin shows reduced collagen types I and III, fragmentation of elastic fibers, slower epidermal turnover and flattening of the dermo-epidermal junction (Gao et al., 2023). Fibroblast activity diminishes, MMPs become upregulated and ECM integrity is progressively compromised (Bocheva et al., 2021). The basement membrane weakens, while impaired lipid synthesis and disrupted tight junctions increase transepidermal water loss (Gao et al., 2023).

Immunologically speaking, aging impairs both innate and adaptive responses. Macrophages, dendritic cells, mast cells and lymphocytes exhibit senescence-related changes including reduced cytokine production, impaired antigen presentation and diminished responsiveness. Increased regulatory T cells suppress protective immune signalling, contributing to an immunosuppressive skin environment (Bocheva et al., 2021; Lutshumba et al., 2021). These deficits weaken the skin’s ability to fight infection, heal wounds and regulate inflammation (Rocamora-Reverte et al., 2021). A key feature of aging skin is “inflammaging,” a state of chronic, low-grade inflammation driven by the accumulation of senescent cells (Baechle et al., 2023). Environmental factors exacerbate these processes. Ultraviolet radiation, particularly UVA and UVB, accelerates oxidative damage, DNA mutations, lipid peroxidation and MMP activation via activation of mitogen-activated protein kinase (MAPK) and AP-1 pathways (Wei et al., 2024). Airborne pollutants, acting through aryl hydrocarbon receptor (AhR) signalling, further stimulate the release of inflammatory mediators and oxidative stress pathways, aggravating ECM breakdown and immunological dysfunction. Together, these structural, molecular and immune alterations compromise skin integrity and repair capacity. Table 2 outlines current interventions and their mechanisms of action in managing skin aging (Shin et al., 2023).

**TABLE 2:** Management strategies for dermal aging and their mechanisms of action (Shin et al., 2023).

Strategy	Approach	Action mechanism	Dermal effects	Advantages	Limitations
Photoprotection	- Broad-spectrum sunscreen - UV filters	- Blocks UVA/UVB radiation - Reduces ROS - Inhibits MMP activation	- Prevents collagen breakdown - Preserves ECM integrity	- Non-invasive - Preventive	- Requires strict compliance - Does not reverse damage
Laser therapy	- CO_2_, Er:YAG (ablative) - Fractional lasers	- Thermal injury induces wound healing cascade - Increases TGF-β and collagen synthesis	- Stimulates fibroblasts - Remodels collagen matrix	- Effective remodeling - Long-term benefits	- Downtime (ablative types) - Risk of PIH in darker skin
	Picosecond lasers	Photomechanical stress enhances fibroblast activity without epidermal damage	- Promotes neocollagenesis - Minimal downtime	Safe for most skin types	Limited penetration depth
High-intensity focused ultrasound	Focused ultrasound beams	Induces thermal coagulation zones in dermis and SMAS layers	- Collagen contraction - Neocollagenesis - Improved elasticity	- Non-invasive - No downtime	- Pain - Operator-dependent outcomes
Radiofrequency (RF)	Monopolar, bipolar or fractional RF	Dermal heating triggers fibroblast activation and ECM remodeling	- Increases collagen/elastin synthesis - Improves laxity	- Suitable for all skin types - Minimal downtime	Multiple sessions often needed
RF microneedling	RF + microneedles	Controlled dermal injury plus RF energy stimulates deeper collagen remodeling	- Enhanced dermal penetration - Collagen realignment	Synergistic efficacy	Potential discomfort and erythema
Light-emitting diodes (LEDs)	Red/near-infrared LED therapy	- Photobiomodulation via mitochondrial stimulation - Reduces inflammation	Stimulates collagen production, supports healing	Safe, affordable and non-invasive	Requires multiple sessions with mild results
Topical retinoids	Retinol, tretinoin	- Binds nuclear receptors, increases epidermal turnover - Enhances collagen synthesis	- Smooths wrinkles - Thickens dermis	Widely studied and considered gold-standard anti-aging	Irritation and photosensitivity
Topical antioxidants	Vitamin C, E, niacinamide	- Neutralize ROS - Reduce oxidative stress - Protect ECM proteins	- Prevents MMP-mediated degradation - Enhances barrier repair	Synergistic with sunscreen	Stability and penetration issues
Topical peptides	- Matrixyl - Copper peptides	- Mimic ECM fragments - Signal fibroblast activity	Stimulate collagen, elastin and glycosaminoglycan production	Good tolerability	Slower onset of action
Hydrating agents	- Hyaluronic acid - Glycerol	- Attract and retain water in ECM - Improve turgor	Plumps skin and reduce wrinkle visibility	Immediate aesthetic benefit	Transient effects with no structural remodeling

## 3 Wounds and their classifications

**FIGURE 4:**
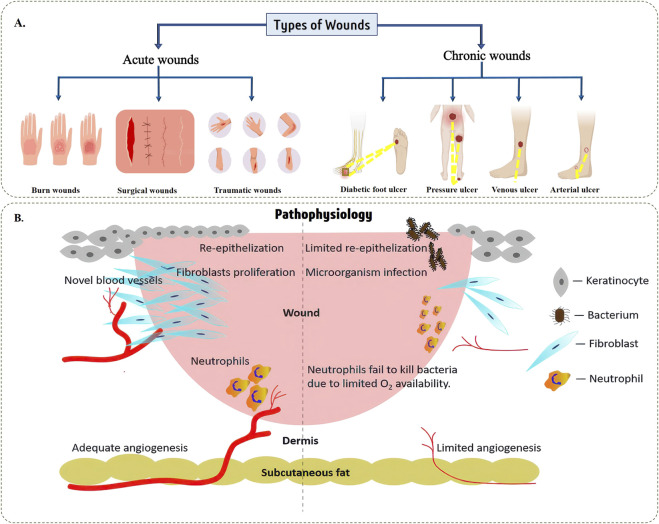
**(A)** A brief overview of different wounds (Joshi et al., 2025). **(B)** Pathophysiology of acute and chronic wound (Wang et al., 2020). Reprinted with permission (Joshi et al., 2025; Wang et al., 2020).

Physical injuries that compromise the integrity of the skin, underlying tissues or mucous membranes are collectively categorized as wounds (Nguyen et al., 2023). Clinically, wounds are classified into acute and chronic based on the duration and nature of the healing process as illustrated in Figure 4. Acute wounds follow a predictable healing course, typically resolving within 4 weeks. In contrast, chronic wounds fail to progress through the normal phases of healing and often remain in a prolonged inflammatory state (Kolimi et al., 2022). Common causes of sustained inflammation include persistent microbial biofilms, hyperglycaemia and advanced glycation end-products (in diabetic ulcers), repeated mechanical ischemia (in pressure ulcers), impaired macrophage M1-to-M2 transition and protease-Tissue Inhibitor of Metalloproteinases (TIMP) imbalance leading to excessive ECM degradation (Kolimi et al., 2022). These mechanisms are discussed in detail in Sections 4 and 5.

### 3.1 Acute wounds

Acute wounds result from sudden tissue injury and typically progress through the orderly stages of hemostasis, inflammation, proliferation and remodeling. When managed appropriately, most heal within weeks. They are broadly categorized into burn, surgical and traumatic wounds. Although acute wounds generally follow a predictable repair trajectory, infection, excessive inflammation or systemic comorbidities may delay healing and predispose them to chronicity (Ding et al., 2022; Sussman, 2023).

#### 3.1.1 Burn wounds

Burn injuries are the most common type of acute wounds and are usually superficial. Burns result from thermal, chemical, electrical or radiation exposure and are associated with high morbidity and mortality worldwide, causing ∼180,000 deaths per year (Ding et al., 2022). Superficial burns usually involve only the epidermis and heal in 3–6 days without scarring. First aid includes cooling with running water and applying non-adherent or antimicrobial dressings, but care should be taken to not use ice, as temperatures below 5 °C can deepen the burn (Sussman, 2023). Deeper burns damage the dermis or subcutis and are associated with complications such as scarring, contractures, pain and psychological stress.

#### 3.1.2 Surgical wounds

Surgical site wounds are the next predominant type of acute wounds. Incisions formed through the skin and underlying tissues during a surgical treatment are referred to as surgical site wounds (Seidelman et al., 2023). To reduce tissue damage, these wounds are often made in a controlled environment with neat, distinct edges. Surgical wounds can be deeper, where the incision passes into muscle and other tissues that may also need to heal or they may be superficial, when just the skin and subcutaneous tissue are damaged (Schimmel et al., 2020). However, recovery can be maximized and the chances of complications (dehiscence or scarring) decreased with proper post-operative treatment, which includes wound surveillance, hydration and appropriate dressings. Recent advancements in surgical wound management include antibacterial-coated sutures, hydrocolloid or foam-based dressings and antimicrobial hydrogels that reduce microbial colonization (Nandel and Saini, 2007; Ding et al., 2022; Seidelman et al., 2023).

#### 3.1.3 Traumatic wounds

Traumatic wounds arise from accidents, blunt force or penetrating injuries and are often irregular in shape, contaminated and are accompanied by variable degrees of tissue damage (Grada and Phillips, 2022). These wounds present significant challenges due to risk of infection, foreign body retention and unpredictable healing (Las Heras et al., 2024). In order to prevent infection, it is usually advised to thoroughly and promptly clean the traumatic wounds. Depending on the nature and extent of the damage, wound closure may then be necessary (Grada and Phillips, 2022). Key advancements in this area include biological scaffolds, 3D-printed dermal templates and skin substitutes that promote dermal regeneration while preventing microbial infiltration. (Falanga et al., 2022).

### 3.2 Chronic wounds

Chronic wounds are lesions that fail to progress through the normal phases of healing and typically persist beyond 3 months (Falanga et al., 2022). Unlike acute wounds, which follow a coordinated sequence of hemostasis, inflammation, proliferation and remodeling, chronic wounds become arrested in a sustained inflammatory state. They are frequently associated with systemic conditions such as diabetes, vascular insufficiency or prolonged immobility (Gefen and Soppi, 2020; Raffetto et al., 2021; Shiffman, 2020). Despite their differences, chronic wounds share several pathophysiological features: sustained inflammation, impaired angiogenesis, excessive protease activity, presence of senescent cells and persistent microbial biofilms. Effective management requires an integrated approach that includes infection control, optimization of systemic conditions, debridement and advanced wound therapies. Emerging strategies involve bioengineered skin substitutes, growth factor delivery, stem cell therapy and smart wound dressings designed to address the biological complexity of chronic wounds (Las Heras et al., 2020).

#### 3.2.1 Diabetic foot ulcers

**FIGURE 5:**
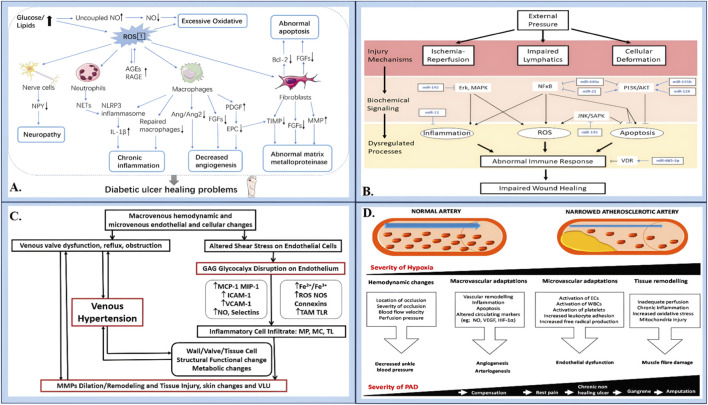
Interplay between different messengers involved in **(A)** diabetic foot ulcer (Liu et al., 2021), **(B)** pressure ulcer (Niemiec et al., 2021), **(C)** venous ulcer (Raffetto et al., 2021) and **(D)** arterial ulcer (Krishna et al., 2015). Reprinted with permission (Liu et al., 2021; Niemiec et al., 2021; Raffetto et al., 2021; Krishna et al., 2015).

According to the World Health Organization, diabetes mellitus affects an estimated 422 million people globally and is directly responsible for approximately 1.5 million deaths annually (Duncan et al., 2026). Diabetic foot ulcers are a common and serious complication of uncontrolled diabetes, affecting approximately 15% of diabetic patients. These ulcers typically develop on the plantar surface of the foot due to a combination of neuropathy, ischemia and mechanical pressure and may extend to deeper structures such as tendons or bone (El-Ashram et al., 2021; Raja et al., 2023). These ulcers mostly affect the lower extremities, especially the foot. The soft tissues, joints and bones of the foot and ankle are often severely damaged as a result. From a clinical perspective, ulcerated diabetic skin has symptoms like those of normal wounds, such as redness, swelling and pus production, but it is often warmer, generates more exudate and frequently smells worse. Systemic infections, severe cellulitis that extends more than 2 cm beyond the wound edge, bone necrosis, gangrene and a decreased oxygen supply to the afflicted limb are all possible signs of advanced stages (Liechty et al., 2020). Hyperglycemia contributes to delayed healing by promoting the accumulation of advanced glycation end-products (AGEs), which induce oxidative stress and chronic inflammation (Figure 5A). Elevated levels of TNF-α, IL-1β and MMPs degrade extracellular matrix components, while reduced angiogenesis and fibroblast dysfunction impair tissue regeneration (Liechty et al., 2020). At the molecular level, several signalling pathways contribute to impaired healing in diabetic foot ulcers (Aljohary et al., 2024). Hyperglycaemia activates the polyol and hexosamine pathways and raises diacylglycerol levels, which in turn activate protein kinase C (PKC). PKC activation promotes NF-κB-driven expression of pro-inflammatory cytokines (TNF-α, IL-1β, IL-6) and suppresses insulin signalling via IRS-1 serine phosphorylation. AGE binding to RAGE further amplifies NF-κB activation and oxidative stress through NADPH oxidase. In parallel, impaired PI3K/Akt and eNOS signalling reduce nitric oxide availability, limiting vasodilation and angiogenesis. HIF-1α stabilization, which normally drives VEGF-mediated angiogenesis under hypoxia, is blunted in diabetic wounds due to methylglyoxal-induced degradation. Together, these pathways explain the persistent inflammation, defective angiogenesis and delayed closure that characterize diabetic foot ulcers (Liechty et al., 2020; Dawi et al., 2025).

#### 3.2.2 Pressure ulcers

Pressure ulcers or pressure injuries, are localized skin and tissue lesions caused by sustained mechanical forces such as pressure, shear or friction, often over bony prominences like the sacrum, heels and hips or from prolonged contact with medical devices (Kottner et al., 2020; Sardo et al., 2023). The elderly, particularly those with limited mobility, incontinence or malnutrition, are at highest risk. Pressure ulcers are associated with high morbidity and mortality, with over 60,000 deaths annually in the US and healthcare costs exceeding $11 billion (Afzali Borojeny et al., 2020; Niemiec et al., 2021). Pathogenesis involves ischemia-reperfusion injury, oxidative stress and mitochondrial dysfunction as depicted in Figure 5B (Niemiec et al., 2021). Persistent pressure disrupts microcirculation, reduces oxygen delivery and triggers cell death pathways. Accumulation of ROS, inflammatory cytokines and MMPs contributes to chronic inflammation and matrix degradation. These wounds often present with undermined edges, slough and signs of infection (Van Damme et al., 2020). Apoptosis and the breakdown of the ECM result from cellular deformation brought on by prolonged or repetitive mechanical pressures, which also damages membrane integrity, increases calcium influx and produces ROS (Van Damme et al., 2020). These processes sustain a persistent inflammatory condition marked by heightened infiltration of neutrophils and macrophages, raised levels of pro-inflammatory cytokines like TNF-α and IL-6 and enhanced activity of MMPs, all of which impede wound healing and tissue regeneration (Liechty et al., 2020). These processes are made worse by immune dysregulation, which is especially prevalent in diseases like diabetes and contributes to the persistence of pressure ulcers (Liechty et al., 2020).

#### 3.2.3 Venous ulcers

Venous leg ulcers (VLUs) are the most common form of chronic leg ulcers, accounting for 70%–90% of all cases, typically resulting from chronic venous insufficiency (CVI) (Raffetto et al., 2021; Stanek et al., 2023; Wang et al., 2023). Impaired venous return leads to sustained venous hypertension, capillary leakage and tissue damage, especially in the gaiter region around the medial and lateral malleoli. Clinically, VLUs are associated with leg edema, varicose veins, skin hyperpigmentation and lipodermatosclerosis (Raffetto et al., 2021; Mayrovitz et al., 2023). These ulcers are slow to heal. Up to 50% remain open beyond 6–12 months and around 10% persist for over 5 years (Raffetto et al., 2021; Stanek et al., 2023).

The pathogenesis of VLUs involves multiple overlapping mechanisms. Venous reflux or obstruction elevates venous pressure and disrupts microcirculation. Impaired calf muscle pump function and limited ankle mobility further contribute to blood pooling and local hypoxia (Figure 5C) (Stanek et al., 2023). At the cellular level, altered shear stress activates endothelial cells to release proinflammatory mediators such as TNF-α, IL-1 and TGF-β1 and increases expression of adhesion molecules like ICAM-1 and VCAM-1 (Raffetto et al., 2021). This triggers leukocyte infiltration, oxidative stress and persistent inflammation. MMPs are upregulated, degrading extracellular matrix and impairing tissue remodeling. Other factors, including fibrin cuff formation, reduced oxygen diffusion and sequestration of growth factors, further hinder wound repair (Wang et al., 2023).

#### 3.2.4 Arterial or ischemic ulcers

Arterial ulcers, also known as ischemic ulcers, are chronic wounds resulting from inadequate blood flow due to peripheral arterial disease (PAD). They account for approximately 5%–20% of non-healing lower limb ulcers, predominantly affecting the elderly population (Las Heras et al., 2020; Falanga et al., 2022). Common risk factors include atherosclerosis, hypertension, diabetes and atrial fibrillation. These ulcers arise from localized ischemia caused by inadequate blood flow to the lower extremities, which fails to fulfill tissue nutrient and metabolic needs (Hoversten et al., 2020).

Arterial ulcer is clinically different from venous ulcer in a number of ways. Venous ulcers often have an uneven form and are shallow and superficial. These ulcers typically manifest on pressure-prone areas like the toes and malleolar regions, presenting as well-defined, “punched-out” lesions with minimal granulation tissue. Patients often experience severe pain, especially at night, which can disrupt sleep (Raffetto et al., 2021; Mayrovitz et al., 2023). Healing is challenging without restoring adequate blood circulation and comorbidities often necessitate surgical interventions. Differentiating arterial ulcers from venous ulcers is crucial, as the latter are usually shallow, irregularly shaped and associated with edema and skin changes due to venous insufficiency. A broad overview, comparing and summarizing the wound types discussed so far are detailed in Table 3. Molecular mechanisms involved in their physiology are described in Figure 5.

**TABLE 3:** Comparison of major wound types: etiology, pathology and molecular signatures.

Wound type	Etiology	Histopathology	Cells involved	Key markers	Characteristic features
Acute wounds					
Traumatic wound	Mechanical injury (abrasion, laceration, crush)	Disruption of tissue architecture, hemorrhage, acute inflammation	Neutrophils (early), macrophages (late), keratinocytes	↑ IL-6, IL-8, TNF-α, VEGF, PDGF, TGF-β1	Irregular borders, variable depth, rapid immune activation
Surgical wound	Incision under sterile conditions	Clean margins, minimal necrosis, organized inflammatory response	Platelets, neutrophils, fibroblasts, endothelial cells	↑ PDGF, TGF-β, VEGF, balanced MMP/TIMP levels	Predictable healing, low infection risk
Burn wound	Thermal, chemical or electrical insult	Coagulation necrosis, dermal denaturation, vascular thrombosis	Infiltrating neutrophils, mast cells, fibroblasts	↑ IL-1β, TNF-α, HSPs, ROS, MMPs, prolonged inflammatory cytokines	Risk of fluid loss, infection, hypertrophic scarring
Chronic wounds					
Diabetic foot ulcer	Neuropathy, ischemia, impaired immune function	Epidermal thinning, dermal sclerosis, capillary basement membrane thickening	Senescent fibroblasts, M1 macrophages, impaired keratinocytes	↑ MMP-9, AGEs, IL-1β, TNF-α, ↓ VEGF, impaired HIF-1α response	Painless, deep, often infected; associated with poor glycemic control
Venous leg ulcer	Venous hypertension, valvular incompetence	Pericapillary fibrin cuffs, leukocyte trapping, dermal fibrosis	Neutrophils, macrophages, iron-laden macrophages	↑ MMP-2/9, VEGF, ferritin, hemosiderin, IL-6	Medial ankle predilection, hemosiderin staining
Arterial ulcer	Atherosclerosis, reduced perfusion	Dry necrosis, sharply demarcated edges, dermal ischemia	Minimal inflammation unless infected; ischemic fibroblasts	↓ VEGF, ↑ ROS, ↑ HIF-1α in early phase, impaired revascularization	Painful, distal toes, punched-out appearance
Pressure ulcer	Prolonged pressure, ischemia, shear	Full-thickness skin loss, muscle/bone involvement in advanced stages	Infiltrating neutrophils, macrophages, apoptotic keratinocytes	↑ IL-1β, TNF-α, nitric oxide, cathepsins, ↑ caspase-3 activity	Sacrum, heels, bony prominences; common in immobile patients

### 3.3 Clinically significant, underrepresented special wounds

Most research and treatment approaches in wound care focus on common wound types like skin ulcers or topical wounds. However, there are other less commonly discussed wounds that are still significant in clinical settings. These include wounds caused by radiation, cancer, autoimmune diseases or certain medications. Such wounds often do not heal properly, show different biological behaviours and do not respond well to standard therapies. They can cause serious complications and are especially challenging to manage in patients who are already sick or have weakened immune systems. This section focuses on these underrepresented wounds and explains why they need more attention in research and treatment.

#### 3.3.1 Radiation-induced skin injuries (RSI)

**FIGURE 6:**
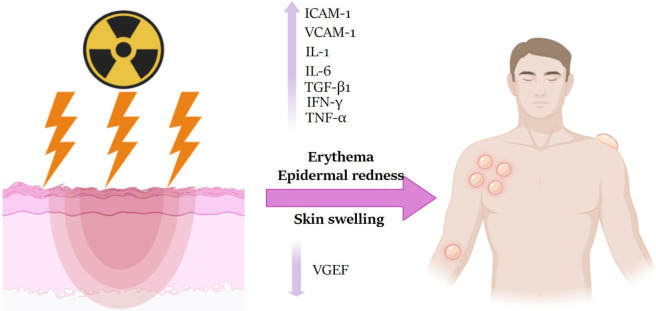
Schematic representation of radiation-induced skin injury, depicting the up- and downregulation of key biomarkers involved in its pathophysiology.

Radiation-induced skin injuries (RSI) are a distinct class of wounds that result from exposure to ionizing radiation, most commonly seen in patients undergoing radiotherapy for cancer (Yang et al., 2020). Unlike mechanical injuries, these injuries present in both acute and chronic phases. Acute effects range from erythema and dry or moist desquamation to ulceration and necrosis, while chronic RSI may lead to epidermal atrophy, fibrosis, telangiectasia and impaired vascularization, features which are hallmark of permanent tissue damage (Bennardo et al., 2021; Chen et al., 2024) (Figure 6).

The underlying mechanisms are complex and multifactorial, driven largely by oxidative stress, chronic inflammation, vascular injury and impaired tissue remodeling. Ionizing radiation produces reactive oxygen and nitrogen species that damage DNA, proteins and lipids, leading to apoptosis of keratinocytes, fibroblasts and endothelial cells. This damage disrupts the skin barrier and activates fibrotic pathways, notably TGF-β, promoting excessive collagen deposition and fibroblast-to-myofibroblast transition (Ryšavá et al., 2021; Cui et al., 2024). Additionally, vascular injury causes tissue hypoxia, impeding nutrient delivery and healing (Rübe et al., 2024). RSI also alters dermal lipid metabolism and immune function. Radiation suppresses epidermal stem cell activity and induces senescence in immune cells, impairing repair and perpetuating inflammation through elevated cytokines like IL-6 and TNF-α (Xu et al., 2022). These combined effects contribute to the chronic, non-healing nature of RSI.

Therapeutic strategies target oxidative damage, inflammation and impaired regeneration. Topical antioxidants (e.g., epigallocatechin gallate), corticosteroids and botanical extracts like chamomile are used to reduce inflammation. Biologic agents such as GM-CSF, EGF and PRP have shown benefits in promoting repair (Lubkowska et al., 2012; Yamakawa and Hayashida, 2019). More advanced therapies, including autologous adipose-derived stem cells (ADSCs) and hyperbaric oxygen therapy (HBOT), are being explored for their ability to improve angiogenesis and reduce fibrosis (Fosen and Thom, 2014). Despite these developments, RSI remains challenging to treat due to the irreversible fibrotic changes and immune suppression caused by radiation. As radiotherapy use continues to grow globally, understanding RSI pathophysiology is critical to developing more effective, targeted interventions.

#### 3.3.2 Electrical burn-associated wounds

Electrical burns represent a unique category of thermal injury caused by the passage of electric current through the body (Potenza, 2023). These injuries can be classified as flash burns, flame burns, lightning strikes or true electrical injuries, the latter involving direct current flow with visible entry and exit wounds. The severity depends on voltage, duration of contact, current pathway and tissue resistance. Although superficial skin damage may appear limited, deeper tissues such as muscles, nerves and blood vessels often suffer extensive, hidden injury. High-resistance tissues like bone can produce heat, increasing the risk of compartment syndrome and deep necrosis. Systemic complications include cardiac arrhythmias, respiratory arrest and acute kidney injury due to rhabdomyolysis. Neurological deficits may occur immediately or be delayed. Management requires rapid cardiovascular stabilization, fluid resuscitation, wound care and monitoring for evolving tissue injury. Despite their complexity and impact, electrical burns remain underrepresented in wound healing research, highlighting the need for greater clinical and mechanistic understanding.

At the molecular and organ level, electrical burns differ from thermal burns in several ways (Żwierełło et al., 2023). Thermal burns produce damage concentrated at the skin surface through direct heat-mediated protein denaturation, with zones of coagulation, stasis and hyperaemia organised around the site of contact. Electrical injury, in contrast, generates Joule heating along the current pathway and causes electroporation, producing pore formation in cell membranes, calcium influx, mitochondrial dysfunction and widespread myocyte and neural necrosis deep within tissues whose surface may appear intact (Tiwari, 2012). Rhabdomyolysis releases myoglobin, which contributes to acute kidney injury through tubular obstruction. Systemic effects are therefore more prominent: cardiac arrhythmias from current passage across the thorax, respiratory arrest, and delayed neurological injury are frequent in electrical burns but uncommon in thermal burns of comparable size. Inflammatory signalling also differs; electrical injury induces sustained elevation of IL-6, TNF-α and creatine kinase, while heat-shock protein (HSP) responses typical of thermal burns are less prominent (Reid and Zhao, 2014). These differences underline why assessment of body surface area alone underestimates the severity of electrical injury and why management prioritizes cardiac monitoring, fluid resuscitation and surveillance for evolving deep-tissue necrosis (Reid and Zhao, 2014).

#### 3.3.3 Chemical burn wounds

Chemical burns result from contact with corrosive agents such as strong acids, alkalis, oxidizers or vesicants (Majeed et al., 2021). These agents damage tissues by disrupting cell membranes and denaturing proteins, leading to coagulative or liquefactive necrosis. Acid burns typically create an eschar that limits spread, while alkali burns penetrate deeper and often cause more extensive damage. The severity depends on the agent’s concentration, contact duration and surface area involved. Immediate and thorough decontamination is critical to prevent further injury. Complications include infection, scarring, delayed healing and systemic toxicity in cases involving chemicals like hydrofluoric acid. These wounds interfere with normal healing by extending inflammation and impairing the function of regenerative skin cells such as keratinocytes and fibroblasts. Due to their varied presentation and potential severity, chemical burns require early recognition, tailored care and more focused research in wound science (Chai et al., 2022).

#### 3.3.4 Autoimmune-associated skin wounds

Autoimmune skin wounds occur when the immune system attacks structural components of the skin, leading to chronic, non-healing lesions (Sun et al., 2024). Conditions such as pemphigus vulgaris, bullous pemphigoid and epidermolysis bullosa acquisita are representative. In pemphigus vulgaris, autoantibodies target desmogleins, causing intraepidermal blistering (Costan et al., 2021). Bullous pemphigoid involves antibodies against basement membrane proteins, leading to subepidermal blistering, while epidermolysis bullosa acquisita targets type VII collagen, resulting in skin fragility. These wounds are painful, slow to heal and highly susceptible to infection. Management relies on systemic corticosteroids and immunosuppressants, although responses vary and prolonged treatment poses additional risks. Their rarity and complexity mean autoimmune skin wounds are often neglected in wound healing research, pointing to a need for more targeted investigation and therapeutic development.

#### 3.3.5 Neoplastic (fungating) skin wounds

Neoplastic or fungating wounds arise from the direct invasion of the skin by malignant tumors (Starace et al., 2022). Commonly associated with advanced-stage cancers such as breast, head and neck and melanoma, these wounds present as ulcerative or nodular growths with necrosis, bleeding, exudate, malodor and pain. The breakdown of tumor vasculature leads to hypoxia and tissue degradation, aggravated by microbial colonization (Niculescu et al., 2024).

Infections worsen inflammation, increase odor and delay healing, significantly impairing patient quality of life. Management is palliative and multidisciplinary, aiming to control symptoms and reduce tumor burden when possible (Fang et al., 2024). This includes specialized dressings for odor and fluid control, pain management and adjunct oncologic therapies. Despite their clinical significance, fungating wounds are understudied, underlining the need for focused research to optimize care and improve outcomes (Starace et al., 2022).

#### 3.3.6 Chemotherapy-induced cutaneous ulcers

Chemotherapy-induced cutaneous ulcers occur when cytotoxic drugs leak into surrounding tissues during intravenous administration, leading to local tissue damage. Severity depends on whether the drug is an irritant or vesicant. Irritants cause mild inflammation, while vesicants, like doxorubicin, can bind DNA and induce prolonged necrosis. Clinically, these ulcers present with pain, swelling and blistering at the infusion site, potentially progressing to deep tissue injury and delayed healing (Sanmartín et al., 2019). Some patients develop linear necrosis along the vein, with persistent erythema and hyperpigmentation (Wyatt et al., 2006).

Early recognition is essential. Immediate interventions include stopping the infusion, aspirating the drug and applying compresses (cold for irritants, warm for specific agents like vinca alkaloids) (Wyatt et al., 2006). Specific antidotes, such as dexrazoxane for anthracyclines, can limit tissue damage if administered promptly. Severe cases may require surgical debridement or grafting. Preventive strategies include careful IV site selection, proper administration techniques and patient education. These ulcers, while preventable, can significantly impair treatment outcomes and patient wellbeing if not managed appropriately (Sanmartín et al., 2019).

## 4 Physiology of wound healing

**FIGURE 7:**
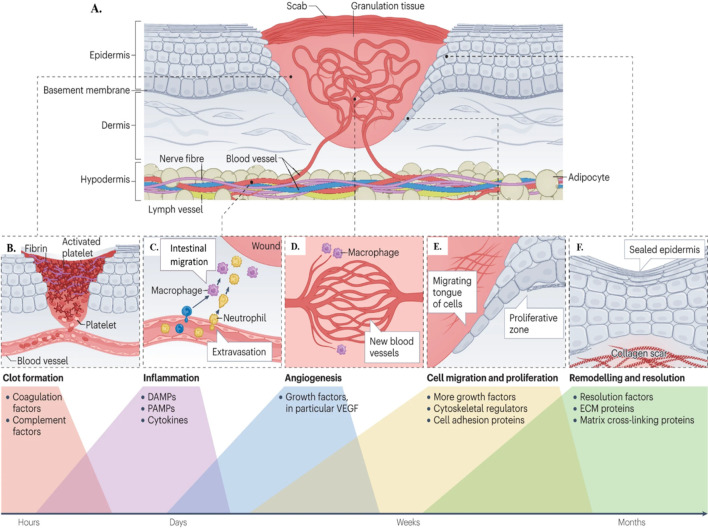
Phases of wound healing (Xiao et al., 2026). Schematic of a skin wound as it closes to illustrate the various phases and contributing cell players **(A)**. Clot formation occurring immediately after tissue damage to temporarily plug wound gap **(B)**. Initiation of inflammatory response cascade involving, innate immune cells, neutrophils and macrophages **(C)**. Macrophages aid the formation of granulation tissue for replacing missing connective tissue, initiating wound angiogenesis **(D)**. Re-epithelialization involving cellular migration and collagen matrix deposition to restore barrier integrity **(E)**. Tissue remodelling to complete the final wound healing stage. DAMPs: damage-associated molecular patterns; PAMPs: pathogen-associated molecular patterns. Reprinted with permission (Xiao et al., 2026).

Following the pathological framework in Section 3, this section describes the molecular and cellular mechanisms of a normal wound repair process. These events provide a reference against which the dysregulation seen in chronic wounds can be evaluated. The orderly progression below is precisely what fails in chronic wounds, where specific checkpoints do not transition. The physiological process of wound healing is an intricately coordinated process and involves four interconnected phases, which include *hemostasis, inflammation, proliferation* and *tissue remodeling* (Raziyeva et al., 2021; Berthiaume Fox et al., 2026). To repair an injured tissue’s integrity and functionality, these stages cooperate in a planned order to restore the body’s hemostasis and architecture (Figure 7). The healing period for normal skin wounds is one to 2 months (Maheswary et al., 2021). Acute injury initiates a coordinated cascade of cellular and molecular events involving growth factors, cytokines and extracellular matrix (ECM) remodelling (Wilkinson and Hardman, 2020). Despite the fact that each phase plays a unique role, they all overlap, highlighting how dynamic and intricate wound healing is.

### 4.1 Phase I—hemostasis

Hemostasis occurs immediately after injury and aims to prevent blood loss and re-establish vascular integrity (Wilkinson and Hardman, 2020). Platelets adhere to exposed collagen in damaged tissue, aggregate and initiate coagulation through intrinsic and extrinsic pathways. This leads to thrombin-mediated conversion of fibrinogen into fibrin, forming a stable clot that temporarily seals the wound (Maouia et al., 2020). Beyond clot formation, activated platelets release cytokines, growth factors and mediators such as ADP, serotonin, von Willebrand factor and prostaglandins, which reinforce vasoconstriction and initiate immune cell recruitment (El-Ashram et al., 2021). The clot also serves as a provisional ECM scaffold. Once bleeding is controlled, fibrinolysis gradually restores vascular patency while chemotactic signals attract inflammatory cells, transitioning to the next phase (Almadani et al., 2021).

### 4.2 Phase II—inflammation

The inflammatory phase is characterized by rapid recruitment of neutrophils, monocytes, macrophages and lymphocytes in response to damage-associated molecular patterns (DAMPs), including HMGB1, heat-shock proteins, S100 proteins, mitochondrial DNA and ATP released from injured cells (Howlader and Das, 2026). These DAMPs bind pattern-recognition receptors such as TLR2, TLR4 and RAGE on resident immune cells, triggering NF-κB-mediated release of chemokines (CXCL1, CXCL8, CCL2) that drive leukocyte infiltration (Kolimi et al., 2022). This stage serves to eliminate pathogens and cellular debris while initiating regenerative signaling. Neutrophils are the first responders and dominate within the initial 24 h. They perform antimicrobial functions and release reactive oxygen species and cytokines. Monocytes subsequently differentiate into macrophages, which orchestrate repair by clearing debris and secreting growth factors such as transforming growth factor (TGF), fibroblast growth factor (FGF), platelet-derived growth factor (PDGF) and epidermal growth factor (EGF); IL-1, IL-6 and IL-17; and ROS (Kolimi et al., 2022). In order to further ingest and digest microbial contaminants, various white blood cells migrate via the circulation to the wound site during the inflammatory phase (Almadani et al., 2021). Increased vascular permeability facilitates immune cell infiltration. Late-stage inflammation is characterized by neutrophil death and a change in macrophage morphologies from pro-inflammatory (M1 phagocytic) to anti-inflammatory (M2 pro-regenerative) (Mahmoud et al., 2024). This transition is essential for progression to proliferation. Growth factors released during this phase recruit fibroblasts, keratinocytes and endothelial cells, priming the wound for tissue reconstruction (Las Heras et al., 2020).

### 4.3 Phase III—proliferation

The proliferative phase focuses on tissue replacement and vascular restoration (Ren et al., 2022). Fibroblasts migrate into the wound bed and synthesize ECM components including collagen, proteoglycans and hyaluronic acid, forming granulation tissue (Mahmoud et al., 2024). Concurrently, endothelial cells drive angiogenesis, restoring oxygen and nutrient supply Keratinocyte proliferation and migration enable re-epithelialization, re-establishing the epidermal barrier. During this stage, coordinated cellular interactions promote matrix deposition, neovascularization and progressive wound contraction. As granulation tissue matures, collagen synthesis strengthens the wound matrix (Nurkesh et al., 2020).

### 4.4 Phase IV—tissue remodelling

Remodeling or maturation, begins approximately two to 3 weeks after injury and may continue for months depending on wound severity (Tottoli et al., 2020). This phase restores tensile strength through ECM reorganization. Type III collagen is gradually replaced by stronger type I collagen and fibroblasts differentiate into myofibroblasts to facilitate wound contraction (Fu et al., 2020; Gushiken et al., 2021). MMPs and their inhibitors regulate balanced ECM turnover, while excess vasculature regresses (Ren et al., 2022).

Growth factors including TGF-β, PDGF and VEGF continue to modulate matrix remodeling and epithelial restoration (Kolimi et al., 2022). Despite successful repair, scar tissue typically recovers only 70%–80% of original tensile strength and lacks appendages such as hair follicles and sweat glands (Tottoli et al., 2020).

### 4.5 Scarring of the skin

Scar formation reflects incomplete architectural regeneration. Fibrotic tissue primarily consists of densely packed type I and III collagen arranged in disorganized or parallel bundles, lacking the complexity of normal dermis (Peña and Martin, 2024). Scar tissue usually contains either aligned, patternless collagen fibers or collagen fibers that are disordered. Persistent inflammation and excessive fibroproliferation contribute to pathological scarring, including hypertrophic scars and keloids, which may extend beyond the original wound boundaries. Functional deficits often arise due to loss of adnexal structures and altered mechanical properties (Peña and Martin, 2024).

## 5 Factors impairing wound healing

Although the body possesses remarkable tissue regeneration mechanisms, a variety of internal and environmental factors can severely disrupt this vital process. In addition to delaying the healing process, these disturbances may result in infections, persistent wounds and other associated complications. Both local causes, such as infections or insufficient blood flow and systemic variables, such as underlying comorbidities, lifestyle choices and age-related changes, can cause such impairments. Identifying and understanding these challenges is crucial to successful healing and incorporating focused treatment approaches into clinical practice.

### 5.1 Nutritional factors

Adequate nutrition is fundamental to wound healing, as it provides the energy and substrates required for immune activation, collagen synthesis and tissue regeneration (Ali, 2024). Deficiencies in macronutrients or micronutrients are strongly associated with delayed healing and increased infection risk (Penny et al., 2022; Munoz and Litchford, 2024).

Protein demand may increase up to 250% during active healing, supporting fibroblast proliferation, angiogenesis and collagen deposition. Carbohydrates provide ATP necessary for cellular metabolism and matrix synthesis, while lipids maintain membrane integrity and support neural insulation (Ghaly et al., 2021; Munoz et al., 2022). Among amino acids, arginine and glutamine are particularly critical. Arginine, a precursor of nitric oxide, supports inflammation, vasodilation and collagen formation. Glutamine enhances immune competence and antioxidant defense (Saeg et al., 2021). Vitamins A, C and D function as essential enzymatic cofactors. Vitamin A regulates immune responses, vitamin C is indispensable for collagen maturation and vitamin D influences keratinocyte proliferation and immune modulation (Munoz and Litchford, 2024). Minerals such as zinc, iron and selenium further support enzymatic reactions, fibroblast function and oxidative balance. Zinc plays a central role across all healing phases by promoting epithelialization and immune. In malnourished or critically ill patients, individualized nutritional assessment and supplementation significantly improve healing outcomes and reduce complications (Penny et al., 2022).

### 5.2 Microbial infections

**FIGURE 8:**
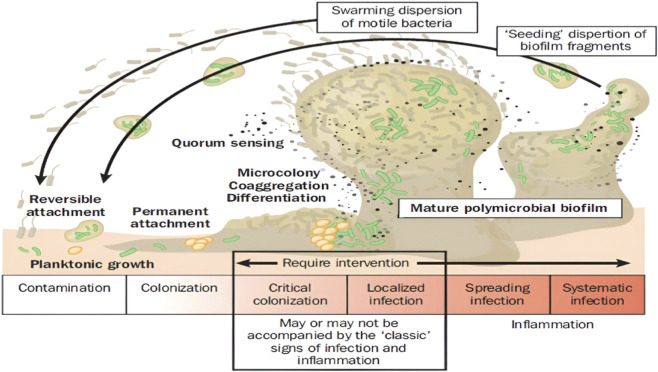
Stages of wound infection continuum depicting impaired wound healing. Reprinted with permission (Vivcharenko et al., 2023).

Infection is one of the most significant barriers to effective wound healing. Microbial colonization disrupts the regulated repair cascade by prolonging inflammation, impairing cellular activity and increasing tissue destruction. This substantially raises morbidity and healthcare costs, particularly in the era of multidrug-resistant organisms (Wynn, 2021). The wound infection continuum includes contamination, colonization and overt infection (Li et al., 2021). The presence of microorganisms in an exposed wound is known as wound contamination (Figure 8).

Early contamination involves the presence of microorganisms without host response. As microbial load increases, localized infection develops, potentially progressing to systemic involvement and sepsis if untreated (Vivcharenko et al., 2023). Microbial infection affects each wound healing phase differently. In hemostasis, bacterial byproducts interfere with clot formation by inhibiting platelet aggregation and degrading fibrin, delaying wound closure initiation (Tottoli et al., 2020; Liang et al., 2021). During inflammation phase, infection amplifies the immune response, with excess pro-inflammatory cytokines (e.g., IL-1, TNF-α) and MMP activity leading to tissue degradation and prolonged inflammation (Caldwell, 2020). Additionally, toxins and biofilms impair neutrophil and macrophage function, preventing efficient bacterial clearance (Vivcharenko et al., 2023).

In the proliferative phase, pathogens inhibit endothelial cell migration and angiogenesis by downregulating growth factor receptors. Biofilms further disrupt fibroblast activity, epithelialization and granulation tissue formation, locking wounds in a chronic state (Liarte et al., 2020; Wynn, 2021). In the final remodeling phase, bacterial toxins and proteases continue degrading collagen and extracellular matrix proteins, impairing scar formation and tensile strength recovery (Nurkesh et al., 2020; Wynn, 2021; Vivcharenko et al., 2023).

### 5.3 Pharmacological impacts on wound healing

Medications can either support or impair wound repair. Certain drugs interfere with cell proliferation, angiogenesis, immune signaling and coagulation, thereby disrupting specific healing phases (Appoo et al., 2024; Bennett et al., 2024). Chemotherapeutic agents are particularly detrimental as they target rapidly dividing cells, including fibroblasts, keratinocytes and endothelial cells. This results in reduced ECM synthesis, impaired angiogenesis and delayed epithelialization (Sharma et al., 2025; Bennett et al., 2024). Agents such as cyclophosphamide and 5-fluorouracil derivatives suppress neovascularization and may induce tissue necrosis or ulcerative complications such as hand-foot syndrome (Zawrzykraj et al., 2023; Bennett et al., 2024).

Nonsteroidal anti-inflammatory drugs (NSAIDs), inhibit prostaglandin synthesis through COX blockade. Although anti-inflammatory, they may reduce tissue perfusion, platelet function and collagen deposition. Experimental studies suggest agents like indomethacin and parecoxib can decrease tensile strength when administered during early healing (Almadani et al., 2021; Bennett et al., 2024). Anticoagulants such as warfarin and heparin may impair clot stability and, in rare cases, cause warfarin-induced skin necrosis. Antibiotics remain essential for infected wounds but do not enhance healing in non-infected wounds and may impair collagen cross-linking (Beyene et al., 2020). Tetracyclines (e.g., doxycycline, minocycline) suppress pro-inflammatory cytokines (IL-1, TNF-α) and inhibit MMPs, potentially disrupting necessary remodeling processes (Beyene et al., 2020). Furthermore, indiscriminate topical antibiotic use increases resistance without improving outcomes. Recent guidelines recommend limiting their use to confirmed, severe infections under clinical supervision (Bennett et al., 2024).

### 5.4 Wound location and external influences

Anatomical location significantly influences healing dynamics. Wounds over bony prominences such as the greater trochanter, olecranon, calcaneal and ischial tuberosities are vulnerable to pressure-induced ischemia, capillary compression and tissue necrosis (Beyene et al., 2020). Similarly, wounds over joints are subjected to mechanical tension and shear forces that impair closure and promote dehiscence. Immobilization with casts or splints may reduce mechanical stress and support healing in high-mobility regions (Bennett et al., 2024).

## 6 TIME principle for wound assessment

**FIGURE 9:**
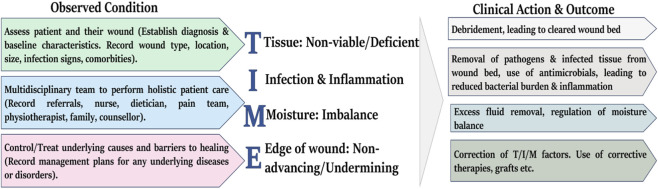
TIME assessment depicting observed condition, the clinical action associated with it and the outcome derived.

The TIME framework provides a structured approach for systematic wound evaluation prior to closure or during secondary healing (Bowers and Franco, 2020). It categorizes wound management into four domains: Tissue (T), Inflammation/Infection (I), Moisture (M) and Edge of wound/Epithelial advancement (E), as depicted in Figure 9 (Alam et al., 2021).

This model has evolved from a wound-focused assessment tool to a broader, patient-centered strategy that integrates systemic disease management with local wound care (Alam et al., 2021). The “T” component emphasizes removal of necrotic tissue, slough, debris and biofilm that impair re-epithelialization. Debridement and wound cleansing are central to restoring a viable wound bed. Adjunctive approaches such as negative pressure wound therapy (NPWT) may assist by reducing edema, improving perfusion and promoting granulation tissue formation (Zens et al., 2020).

The “I” domain addresses persistent inflammation and infection, which elevate protease activity and delay healing. Clinical signs such as redness, warmth, swelling or delayed progression warrant evaluation for microbial burden or biofilm formation (Alam et al., 2021). Targeted antimicrobial therapy, guided by clinical judgment and culture when necessary, is essential to restore physiological repair processes. The “M” category focuses on maintaining balanced moisture. Excess exudate promotes maceration and protease accumulation, whereas desiccation impairs cellular migration (Falanga et al., 2022). Appropriate dressings should therefore regulate fluid levels to support optimal healing conditions (Zens et al., 2020).

Finally, the ‘E’ component evaluates epithelial advancement and wound-edge contraction. Failure of edge progression often indicates unresolved issues in the other TIME domains. In such cases, adjunctive therapies may be considered to stimulate tissue regeneration (Bowers and Franco, 2020). While the TIME framework standardizes clinical assessment, traditional approaches remain limited in providing real-time insights into wound biology. Emerging smart dressings equipped with biosensors and responsive drug delivery systems offer the potential to monitor biomarkers such as pH, temperature and inflammatory mediators, enabling earlier detection of complications and more precise therapeutic intervention (Zens et al., 2020).

## 7 Therapeutic and translational innovations

**FIGURE 10:**
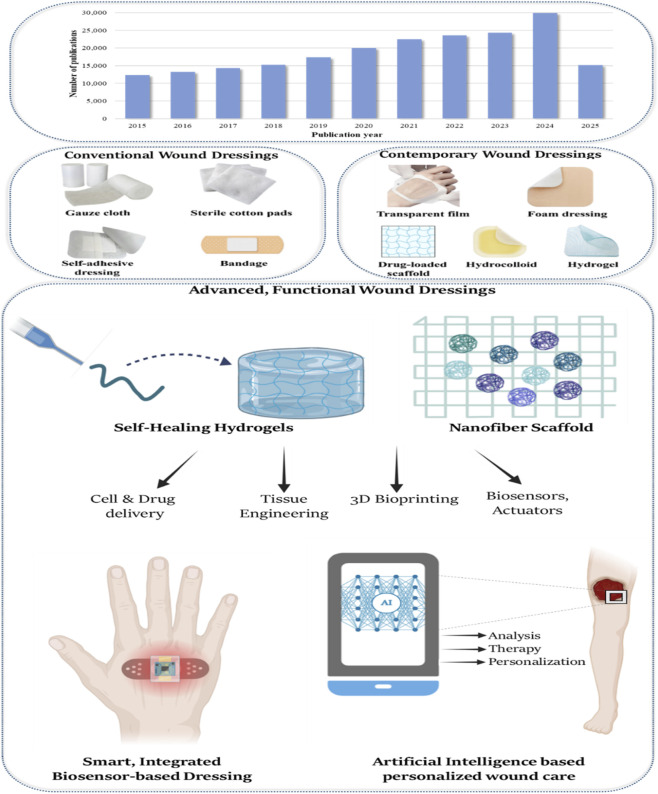
Based on the web of science core collection search results graph shows an increasing trend in research on advanced wound care and management in the last decade (Upper column). The search keywords included “wound care,” “therapeutic strategies,” “advanced wound dressings,” “bioengineered dressings,” “3D bioprinting,” “gene therapy,” “stem cell therapy,” “smart dressings,” “nanofiber scaffolds,” “bioactive dressing,” “biosensor dressing,” “hydrogels,” “AI-driven wound care.” Schematic representation of conventional dressings depicting traditional form of wound care and contemporary dressings depicting modern wound dressings in the middle column (Joshi et al., 2025). Advanced wound dressings illustrating the recently developed dressing trends in the lower column. Owing to their high moisture retention, gas permeability and biocompatibility, SHHDs are particularly suitable for chronic wound management (Zhang et al., 2024). Their injectable and adhesive properties enable effective coverage of irregular wound geometries, while their hydrated 3D networks mimic aspects of native extracellular matrix architecture (Zhang et al., 2023).

Conventional wound dressings, including gauze, hydrocolloids, foams, films and alginate-based materials, are well established for maintaining moisture balance and providing physical protection. As their composition and clinical applications have been extensively reviewed, the present discussion focuses on advanced platforms designed to actively interact with the wound microenvironment. Contrary to conventional or contemporary dressings, advanced wound dressings are combined with polymeric substrates and biosensors to enable real-time monitoring of wound biomarkers (Pang et al., 2023). These dressings are equipped to release bioactive materials such as medications or growth hormones that are incorporated in them or they may possess inherent healing qualities (Vo and Trinh, 2025). While growth factors promote tissue regeneration, the integrated drugs operate as cleaning or debriding agents to eliminate dead tissue or to treat the associated infections (Tiwari and Pathak, 2023). Since they reduce the need for dressing changes and speed up the overall wound healing process, advanced dressings, which incorporate state-of-the-art materials and technology, prove beneficial for managing chronic wounds (Joshi et al., 2026). Figure 10 illustrates the advancements in wound dressings.

### 7.1 Self-healing hydrogel dressings

Self-healing hydrogel dressings (SHHDs) derive their repair capability from reversible chemical or physical interactions within their three-dimensional polymer networks (Figure 10) (Zheng et al., 2021; Yang J. et al., 2023). These reversible dynamic covalent or noncovalent bonds allow the material to recover structural integrity after mechanical disruption (Mishra et al., 2024). At the molecular level, delivery from SHHDs is controlled by the reversible bonds that form the network. Dynamic covalent bonds, such as Schiff base (imine) linkages between aldehyde and amine groups, boronate esters between boronic acids and diols and disulfide exchange, dissociate and reform under physiological conditions. Non-covalent interactions, including host-guest complexation (cyclodextrin-adamantane), metal-ligand coordination (Fe^3+^ with catechol or carboxylate groups) and hydrogen bonding, provide further reversibility (Talebian et al., 2019). These bonds can be engineered to break in response to specific wound cues *viz.* boronate esters dissociate with elevated ROS or glucose, imine bonds hydrolyze at low pH and metal-coordination bonds respond to changes in local redox state (Yin et al., 2024). As a result, bioactive molecules are released in proportion to the inflammatory, oxidative or infectious status of the wound, rather than at a fixed rate.

**FIGURE 11:**
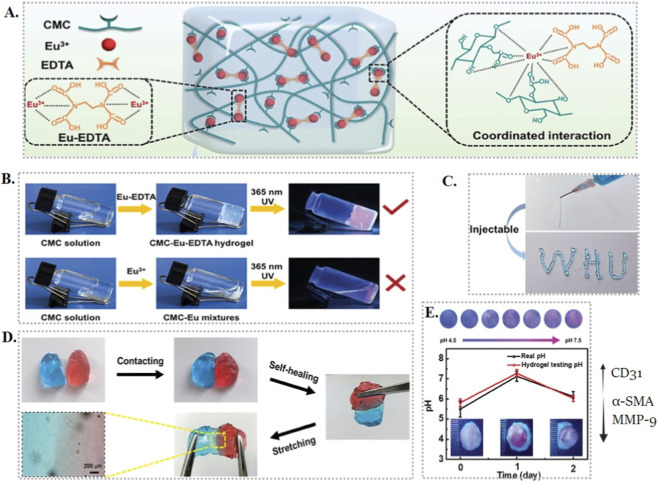
**(A)** SHHD made of carboxymethyl cellulose cross-linked via Eu-EDTA, **(B)** CMC-Eu-EDTA Hydrogel, **(C)** Injectability of developed hydrogel, **(D)** Self-healing and elasticity of SHHD and **(E)** Change in pH affecting gel characteristics. The application of hydrogel *in vivo* upregulated CD31 and downregulated MMP-9 and α-SMA expression. Modified and adapted from Xie et al., (2024).

Recent SHHD designs integrate therapeutic and sensing capabilities. Xie et al. developed a multifunctional hydrogel composed of carboxymethyl cellulose cross-linked through europium-EDTA coordination (Xie et al., 2024). The material demonstrated injectability, rapid self-repair and tissue adhesion, along with pH-responsive fluorescence for non-invasive wound monitoring (Figure 11). *In vitro*, it enhanced endothelial cell proliferation and migration, while in diabetic rat models it promoted angiogenesis, collagen deposition and granulation tissue formation. These effects were associated with reduced inflammation, downregulation of MMP-9 and upregulation of angiogenic markers.

Similarly, ROS-responsive hydrogel based on polyvinyl alcohol (PVA) and a dynamic boronate ester crosslinker, incorporating gelatin methacryloyl microgels loaded with sodium fusidate and metformin hydrochloride (Guo et al., 2025). The system demonstrated sustained drug release, antibacterial activity exceeding 98% within 24 h and effective ROS scavenging. Each component contributes a distinct function. PVA forms the biocompatible backbone that provides hydration and mechanical support (Yang M. et al., 2023). The dynamic boronate-ester crosslinker is the source of ROS responsiveness since elevated ROS in the inflamed wound oxidatively cleaves boronate bonds, softening the network and releasing target molecules on demand (Liang and Liu, 2016). Gelatin methacryloyl (GelMA) microgels embedded in the network act as reservoirs that fine-tune release kinetics and provide cell-adhesive RGD motifs. Sodium fusidate, a bacteriostatic antibiotic targeting bacterial protein synthesis via elongation factor G, drives the >98% antibacterial activity. Metformin contributes through AMPK activation, which suppresses NF-κB signalling, reduces pro-inflammatory cytokine production and promotes angiogenesis in the diabetic wound bed (Guo et al., 2025).

In diabetic rat models, it accelerated wound closure and enhanced vascularization and collagen remodeling. This design illustrates the integration of self-healing, stimuli-responsive and therapeutic functionalities in a single platform. Another approach involved adhesive, conductive and antibacterial SHHDs constructed using oxidized sodium alginate-grafted dopamine/carboxymethyl chitosan/Fe^3+^ networks combined with polydopamine-encapsulated poly (thiophene-3-acetic acid) (Qiao et al., 2023). Dynamic Schiff base and Fe^3+^ coordination interactions enabled structural self-repair. The hydrogel exhibited photothermal antibacterial activity under near-infrared exposure, along with adequate mechanical strength, adhesion and antioxidant properties. In infected full-thickness wound models, treated wounds showed significantly improved closure compared to commercial controls after 14 days.

Overall, SHHDs demonstrate the convergence of structural adaptability, therapeutic delivery and diagnostic responsiveness. While further clinical validation is required, these systems represent an important advancement toward multifunctional wound care platforms.

### 7.2 Nanofiber scaffolds and bioactive dressings

Nanofiber-based scaffolds represent a promising class of bioengineered wound dressings designed to mimic the native ECM, offering structural support and functional cues for tissue regeneration (Jiang et al., 2023). These scaffolds are commonly fabricated via electrospinning, which produces highly porous fibers with large surface-area-to-volume ratios and tunable mechanical properties that support cell adhesion, migration and nutrient diffusion (Behere and Ingavle, 2022; Joshi et al., 2024). Incorporation of bioactive agents such as antimicrobial peptides, growth factors, phytochemicals or nanoparticles enables active modulation of the wound microenvironment (Sharma P. et al., 2022; Guo et al., 2025).

A key advantage of nanofiber dressings lies in their ability to deliver therapeutics in a controlled and sustained manner (Sharma S. et al., 2022; Sharely et al., 2025). Nanofibers loaded with EGF or VEGF have enhanced angiogenesis and granulation tissue formation in diabetic and chronic wound models (Liu et al., 2022; Zhan et al., 2022). Surface functionalization allows responsiveness to pH or reactive oxygen species, enabling stimulus-triggered drug release (Naghib et al., 2024). Biodegradable polymers such as PCL and PLA, as well as natural polymers including chitosan and gelatin, are widely used to ensure biocompatibility and gradual degradation (Dong et al., 2024). Integration of antimicrobial nanoparticles such as silver, zinc oxide or copper further improves infection control in chronic wounds (Alven et al., 2021; Sabarees et al., 2022; Dang et al., 2023). Core–shell nanofibers allow compartmentalized or sequential drug release for enhanced therapeutic precision (Bayer, 2021; Huo et al., 2021; Jiang et al., 2023). Hybrid systems combining nanofibers with hydrogels or sensing elements provide added benefits such as moisture retention, mechanical stability and monitoring capability (Serafin et al., 2021; Abadi et al., 2022; Qiu et al., 2022).

**FIGURE 12:**
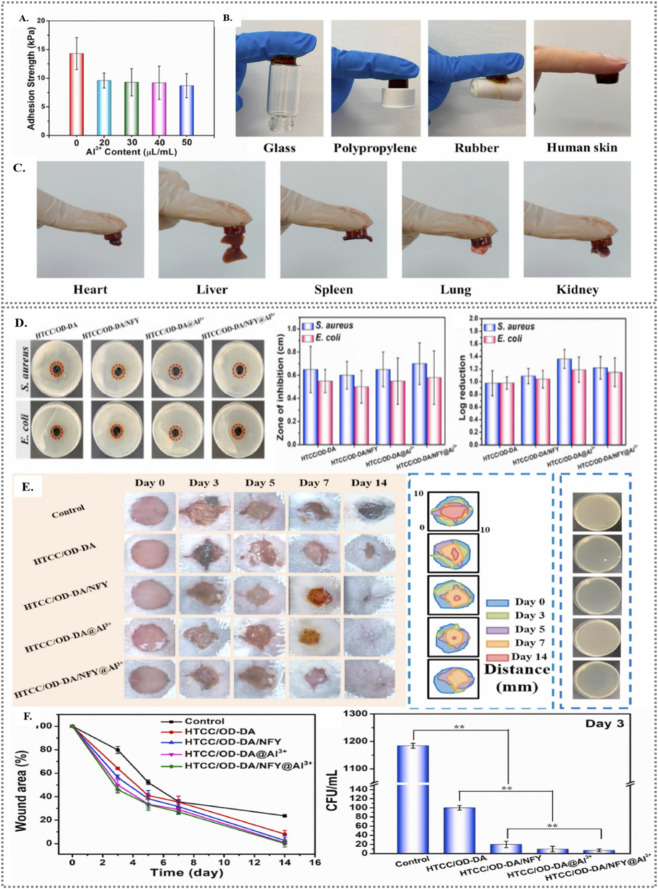
3D hybrid, antibacterial and conductive scaffold (Zhou et al., 2024). **(A)** Adhesion strength of prepared scaffold with porcine skin, **(B, C)** Hydrogels adhered to the surface of various materials and tissues, **(D)** Antibacterial properties, **(E,F)** *In vivo* wound closure capability of the prepared hybrid scaffold. Adapted from Zhou et al., (2024).

A recent electrospun scaffold composed of sulfated hyaluronic acid, collagen and polyurethane demonstrated structural similarity to native skin and promoted a moist, bioactive environment (Zhou et al., 2024). In diabetic mouse models, it accelerated wound closure, enhanced vascularization and improved tissue remodeling (Figure 12).

Another approach involved a conductive 3D hybrid scaffold formed by embedding nanofiber yarn networks into a Schiff base-crosslinked injectable hydrogel. The nanofiber yarns, composed of polyacrylonitrile and reduced graphene oxide, promoted cellular alignment, while the hydrogel provided an ECM-like microenvironment. Incorporation of conductive ions enabled pressure sensing and real-time wound monitoring. Although preclinical findings are promising, clinical translation requires validation of long-term safety, reproducibility and scalable manufacturing. Regulatory considerations are particularly relevant when combining synthetic polymers with bioactive agents. Nonetheless, nanofiber-based bioactive dressings represent a significant advancement in regenerative wound care (Yan et al., 2024).

### 7.3 Smart dressings and biosensors: integrating diagnostics with therapeutics

**FIGURE 13:**
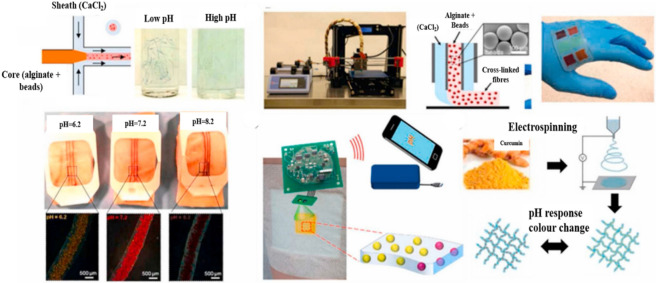
Schematic representation of biosensor-integrated wound dressings and wearable pH-responsive systems showing alginate microfibers with embedded pH-sensitive beads fabricated via microfluidics and integrated into medical tape in the upper panel. The lower panel depicts 3D-printed alginate films containing pH-responsive beads, smart cellulose dressing loaded with GJM534 dye and wirelessly connected to a smartphone for real-time monitoring and electrospun curcumin-loaded fibers. Modified and adapted from Lv et al., (2024).

Smart wound dressings integrated with biosensing technologies represent a shift toward interactive wound management by combining real-time monitoring with therapeutic responsiveness (Youssef et al., 2023; Basu et al., 2024). Unlike conventional or purely bioactive dressings, these systems continuously assess biomarkers such as pH, temperature, glucose, lactate, reactive oxygen species or uric acid and respond to microenvironmental changes (Figure 13) (Lv et al., 2024). Their goal is early detection of infection or inflammatory imbalance and, in some platforms, stimulus-triggered therapeutic release (Pusta et al., 2021).

Most smart systems integrate three components: a sensing module (colorimetric, electrochemical or fluorescent), a responsive polymeric or hydrogel matrix and a therapeutic payload such as antimicrobial or anti-inflammatory agents (Basu et al., 2024). pH-responsive hydrogels, conductive polymers like polyaniline or PEDOT:PSS and ROS-sensitive matrices are commonly used to detect infection-related changes. Advances in wireless transmission, including Bluetooth and NFC interfaces, allow real-time data integration into mobile or clinical platforms. Flexible and stretchable electronics improve conformability without compromising patient comfort. Some designs incorporate feedback mechanisms, such as infection-triggered antibiotic release or temperature-responsive drug delivery (Basu et al., 2024).

As a detailed example, consider a pH-responsive smart dressing used to detect infection. Healthy skin has a pH of 4.0–6.0, whereas infected chronic wounds shift toward an alkaline pH of 7.5–9.0 due to bacterial metabolic byproducts and altered host enzyme activity (Ono et al., 2015; Bennison et al., 2017). Detection uses immobilized pH-indicator dyes (such as bromothymol blue or GJM534) whose protonation state changes with local pH, producing a visible colour change or, in fluorescent systems, a shift in emission wavelength. In electrochemical variants, conductive polymers such as polyaniline undergo pH-dependent protonation of their imine groups. This alters conductivity, which is then transduced into a digital signal through microelectrodes and transmitted wirelessly through NFC or Bluetooth (Mariani et al., 2021). In therapeutic platforms, the same pH shift can trigger drug release by protonating hydrazone or acetal linkages in the hydrogel matrix, releasing encapsulated antimicrobials at the infected site (Das and Bal, 2024). The detection mechanism is therefore directly tied to the chemistry of the polymer network.

These systems are particularly relevant for chronic wounds such as diabetic foot ulcers, pressure injuries and venous leg ulcers, where sustained inflammation and infection require continuous monitoring. Unlike traditional dressings that necessitate removal for assessment, smart dressings enable non-invasive tracking of wound status and may reduce dressing frequency. Multiplexed sensing platforms capable of monitoring multiple biomarkers simultaneously are also emerging (Youssef et al., 2023). Despite promising preclinical results, clinical translation remains challenging. Sensor stability, signal drift and biocompatibility must be ensured for long-term application (Youssef et al., 2023). Manufacturing complexity, cost and regulatory pathways for hybrid drug-device platforms present additional barriers. Power supply and data transmission constraints further limit scalability (Basu et al., 2024).

Integration of artificial intelligence with sensor-enabled dressings may enhance predictive capability by analyzing real-time biomarker data (Kalasin et al., 2022). Convergence with 3D printing and bioresorbable electronics could enable more personalized and programmable systems (Qureshi et al., 2023; Prakashan et al., 2024). Overall, smart dressings represent a transition from passive coverage to responsive wound management, though successful clinical adoption will require technological refinement and interdisciplinary coordination.

### 7.4 Cellular, regenerative and molecular approaches in advanced wound healing

Cellular and regenerative therapies, together with molecular and genetic interventions, represent advanced strategies aimed at directly correcting the biological deficits underlying impaired wound repair (Fani et al., 2024). These approaches are particularly relevant for chronic wounds where endogenous healing responses are insufficient. By leveraging stem cell technologies, bioengineered constructs and gene-based modulation, they seek to enhance regeneration, reduce fibrosis and accelerate closure (Kolimi et al., 2022).

Mesenchymal stem cells (MSCs) and induced pluripotent stem cells (iPSCs) remain central to cellular therapy approaches (Bülbül et al., 2022). MSCs can differentiate into multiple skin-relevant lineages and secrete growth factors, cytokines and extracellular vesicles that modulate inflammation and promote angiogenesis and matrix remodeling (Lashkari et al., 2023). iPSCs offer an expandable autologous cell source with broad differentiation potential, supporting tissue regeneration in complex wounds (Dash et al., 2022). Both have shown encouraging outcomes in preclinical and early clinical studies, particularly in promoting vascularization and epithelial repair (Dash et al., 2022).

Platelet-rich plasma (PRP) is another widely studied therapy, harnessing the regenerative power of platelets, which are rich in growth factors such as VEGF, PDGF and TGF-β (Shi et al., 2021; Zhang et al., 2021; Zhang et al., 2023). PRP has been shown to accelerate wound healing by promoting cell proliferation, migration and matrix deposition (Oneto and Etulain, 2021). Similarly, exosomes have emerged as potent paracrine mediators capable of delivering proteins, lipids and nucleic acids that regulate inflammation and support repair. MSC-derived exosomes, in particular, show strong regenerative effects in wound models (Bakadia et al., 2023). Advances in tissue engineering and 3D bioprinting have enabled the development of biomimetic skin substitutes (Sörgel et al., 2023). These constructs combine keratinocytes, fibroblasts and matrix components to restore barrier function and support re-epithelialization (Chen et al., 2023). Layer-by-layer bioprinting improves structural precision and enables the fabrication of patient-specific grafts that more closely resemble native epidermal and dermal architecture (Tavakoli and Klar, 2021).

On the molecular and genetic front, innovative gene therapy techniques, such as CRISPR-Cas9, are being explored to modulate key signaling pathways involved in wound healing (Shi et al., 2024). For example, CRISPR-Cas9 can be used to edit genes associated with collagen production, TGF-β1 signalling and VEGF expression that are the three critical factors in wound repair (Li et al., 2023). By enhancing collagen synthesis and regulating the inflammatory response, CRISPR-based gene editing holds the potential to accelerate tissue repair and reduce scarring. Gene therapy strategies targeting VEGF, for instance, could promote angiogenesis in ischemic wounds, while modulation of TGF-β1 could help balance fibrosis and regeneration, thereby improving wound healing outcomes (Shaabani et al., 2022; Mullin et al., 2024).

### 7.5 Artificial intelligence-driven personalized wound care

**FIGURE 14:**
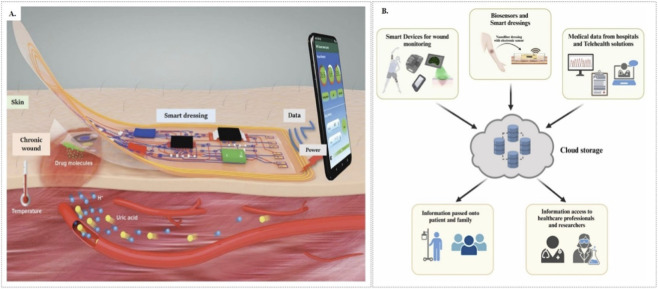
AI-enabled smart wound dressing system for real-time monitoring of chronic wounds, capable of detecting wound biomarkers and transmitting data wirelessly to external devices **(A)**. Integration of smart dressings, wearable biosensors, telehealth systems and cloud-based data storage for remote wound monitoring and clinical decision-making **(B)**. Reprinted with permission (Rezvani Ghomi et al., 2023; Shankhwar et al., 2025).

Artificial Intelligence (AI)-driven personalized wound care is transforming wound management by using advanced computational tools to predict wound progression, optimize treatment strategies and individualize care (Gefen, 2025; Talwar et al., 2024) (Figure 14).

By integrating machine learning (ML), deep learning and decision-support systems into clinical workflows, clinicians can make more informed decisions and potentially improve healing outcomes (Dabas et al., 2023). Machine learning algorithms analyze large datasets including clinical records, wound imaging and patient-specific variables such as comorbidities, age and lifestyle factors (Ayyalasomayajula, 2025). By identifying patterns in historical data, these models can predict healing trajectories and flag risks such as infection, delayed repair or excessive scarring. Early risk identification enables timely intervention and more personalized management strategies.

The integration of AI with wound imaging technologies, such as digital photography, infrared imaging and 3D scanning, enhances the ability to monitor wound changes over time AI integration with wound imaging modalities, including digital photography, infrared imaging and 3D scanning, enhances objective monitoring of wound size, depth and tissue characteristics (Anisuzzaman et al., 2022). Deep learning algorithms can assess wound progression over time and assist in determining whether escalation of care, such as debridement or advanced therapies, is required (Lucas et al., 2021).

A notable example is the a-Heal platform, a portable wireless device recently reported for closed-loop wound management for continuous wound therapy (Li et al., 2025). a-Heal integrates real-time wound imaging, a machine-learning module that classifies healing stage and automated delivery of electric-field stimulation or drug release through a flexible bioelectronic interface. In porcine and murine wound models, a-Heal autonomously identified deviations from the expected healing trajectory, delivered corrective treatment and accelerated tissue regeneration and re-epithelialization compared with standard of care. This illustrates how AI-driven devices can move beyond monitoring toward adaptive, closed-loop therapy in complex wounds (Li et al., 2025). Another innovative example is the iCares platform, which a wearable microfluidic device recently reported for continuous *in situ* analysis of chronic wounds (Wang et al., 2025). iCares integrates a flexible nanoengineered sensor array that measures reactive oxygen and nitrogen species NO, H_2_O_2_, O_2_, pH and temperature, together with pump-free triad microfluidic modules that draw wound exudate to the sensors using a superhydrophobic–superhydrophilic Janus membrane, wedge channels and graded micropillars. Wireless connectivity supports long-term continuous monitoring. In diabetic mouse models, iCares tracked biomarker changes during infection and their resolution after antibiotic treatment. In a cohort of 20 patients with chronic wounds, the multiplexed sensor data were analysed using a machine-learning algorithm, which successfully classified wound severity and predicted healing potential. iCares therefore illustrates how AI-driven devices can move beyond image-based monitoring toward objective, biomarker-informed wound assessment in human patients.

Decision-support algorithms further assist clinicians by recommending treatment strategies based on wound type, patient condition and response to prior therapies (Reifs Jiménez et al., 2025). These systems can guide dressing selection, antimicrobial use and timing of interventions and may support initiation of advanced therapies when indicated (Reifs Jiménez et al., 2025). AI-driven wound care also integrates with wearable devices and smart dressings equipped with biosensors (Prakashan et al., 2024). These platforms monitor biomarkers such as pH, temperature and moisture, transmitting data for real-time analysis (Vo and Trinh, 2025). Predictive models can incorporate underlying conditions, lifestyle factors and vascular status to generate individualized treatment recommendations. Such stratification may support targeted infection control or perfusion-enhancing strategies where appropriate. Improved prediction and early complication detection may also reduce unnecessary interventions and associated healthcare costs (Kuo, 2023). Despite its potential, several challenges limit widespread adoption Model performance depends on high-quality, standardized data. Privacy protection and secure data handling remain essential. Integration into clinical workflows requires clinician training and trust to ensure AI complements rather than replaces clinical expertise (Wang et al., 2024).

A comparative analysis of the discussed advanced wound care therapies have been summarized in Table 4. Future advancements will likely enhance predictive accuracy through larger datasets and improved validation. Integration with regenerative and biomaterial-based therapies may further enable adaptive and personalized wound management.

**TABLE 4:** Comparison of advanced wound care platforms: mechanisms, representative outcomes, advantages, limitations and translational stage.

Platform	Mechanism of action	Representative quantitative outcomes	Key advantages	Current limitations	Translational stage
SHHDs	Reversible dynamic bonds (Schiff base, boronate ester, metal coordination, host–guest); stimulus-responsive release; ECM-mimetic hydrated network	Antibacterial activity >98% within 24 h; ∼70–90% closure at day 14 in diabetic rat models; ↑ CD31, ↑ collagen, ↓ MMP-9	Conform to irregular wound shapes; injectable; retain moisture; support multifunctional integration	Limited mechanical strength; long-term *in vivo* stability not proven; scale-up; few human studies	Preclinical (rodent); limited early-phase clinical
Nanofiber scaffolds and bioactive dressings	ECM-mimetic fibrous architecture via electrospinning; release tuned by fiber morphology and responsive linkers (thioketal, hydrazone); integrin-mediated signals	Accelerated closure in diabetic mice; enhanced vascularization and collagen organization; sequential release from core–shell fibers	High surface-area-to-volume ratio; tunable degradation; supports cell alignment	Some formulations brittle; industrial-scale uniformity; sterilization constraints	Preclinical with early clinical evaluation
Smart biosensor-integrated dressings	Sensing modules (colorimetric, fluorescent, electrochemical) for pH, ROS, glucose, temperature; conductive polymers; NFC/Bluetooth transmission	Detection of pH shifts (0.2–0.5 units) linked to infection onset; multiplexed biomarker readout in preclinical diabetic models	Non-invasive continuous monitoring; fewer dressing changes; compatible with telemedicine	Sensor drift; power and data transmission limits; regulatory complexity; cost	Preclinical and early clinical
MSC/iPSC therapy	Paracrine release of growth factors and cytokines; immune modulation; differentiation into keratinocytes, fibroblasts, endothelial cells	Improved closure and reduced healing time in DFU trials; raised angiogenic and epithelial markers	Multi-lineage potential; broad paracrine secretome; autologous options available	Donor variability; tumorigenic risk (iPSCs); GMP complexity; short shelf-life	Early clinical trials
Exosomes (MSC- and PRP-derived)	Deliver miRNAs (miR-21, miR-126, miR-132), cytokines and lipid mediators; modulate PI3K/Akt, MAPK, Wnt/β-catenin	Enhanced angiogenesis, M2 polarization and collagen remodeling in preclinical diabetic models	Cell-free; lower immunogenicity; stable storage	No standardized isolation; variable potency; regulatory ambiguity	Preclinical, moving to early clinical
PRP	Autologous concentration of platelet growth factors (VEGF, PDGF, TGF-β)	Faster healing in multiple chronic wound indications; reduced wound area in DFU and venous ulcer trials	Autologous; established clinical use; low cost	Variable platelet content; no standard preparation; inconsistent outcomes	Established clinical use
Gene-based interventions (CRISPR-Cas9)	CRISPRa/CRISPRi modulation of VEGF, TGF-β1, collagen pathways	Preclinical only; improved angiogenesis and reduced scarring in mouse models	Pathway-specific; potentially durable	Delivery to wound tissue; off-target edits; evolving regulatory frameworks	Early preclinical
AI-driven personalized wound care	ML and deep-learning models applied to wound imaging, biomarker streams and clinical records	Improved wound assessment accuracy; validated in human 20-patient chronic-wound cohort using integrated sensing + ML	Scalable; compatible with telemedicine	Performance depends on data quality; privacy concerns; regulatory validation	Early clinical adoption

## 8 Future directions and perspectives

The future of wound healing is increasingly aligned with personalized and precision medicine, driven by technological innovation and a deeper understanding of wound biology. Integration of multi-omics platforms, including genomics, proteomics and metagenomics, is expected to refine patient stratification and therapeutic targeting. By characterizing the molecular, cellular and microbial signatures of individual wounds, clinicians may better predict healing trajectories, identify patients at risk of complications and design interventions tailored to specific biological deficits. Such approaches have the potential to reduce chronicity, limit infection and improve functional outcomes.

Predictive biomarkers will further enhance individualized care. Advances in biomarker discovery, combined with AI-assisted analytics, are enabling real-time assessment of inflammatory status, angiogenic potential and tissue remodeling dynamics. These tools may support early identification of delayed healing, guide therapy selection and evaluate treatment response, particularly in complex or chronic wounds. As predictive modeling improves, decision-making is likely to shift from reactive to anticipatory care. Emerging technologies including artificial intelligence, robotics and advanced imaging will continue to reshape wound management. AI-driven systems are expanding beyond monitoring toward predictive modeling and adaptive treatment planning. Robotics may improve precision in procedures such as debridement, enhancing tissue preservation and reducing operator variability. Real-time imaging modalities, including optical coherence tomography and fluorescence imaging, provide non-invasive insights into tissue architecture, perfusion and microbial burden, improving assessment accuracy and longitudinal monitoring.

Skin-on-a-chip platforms represent another promising direction. By recreating human skin architecture within controlled microfluidic systems, these models enable mechanistic studies and preclinical evaluation of drugs, dressings and biomaterials under physiologically relevant conditions. Such platforms may accelerate therapeutic discovery, reduce reliance on animal models and enhance translational relevance by providing more predictive *in vitro* systems.

As innovation accelerates, regulatory and ethical considerations will remain central. Bioengineered constructs, stem cell therapies and gene-based interventions require rigorous clinical validation to ensure safety, durability and reproducibility. Regulatory frameworks must evolve to address hybrid products that combine biological, material and digital components. Ethical oversight is equally important, particularly in gene editing, long-term implantable systems and AI-guided decision-making. Careful evaluation of risk–benefit balance, patient consent and long-term outcomes will be essential to ensure responsible translation of these technologies into clinical practice.

## 9 Conclusion

This review examined the structural organization of the skin, the molecular and cellular mechanisms governing wound repair and the pathophysiological distinctions between acute and chronic wounds. We outlined how effective healing depends on tightly coordinated inflammatory, proliferative and remodeling processes and how dysregulation at any stage can drive chronicity and tissue degeneration. Given the rising global burden of non-healing wounds, there is a pressing need for biologically informed and technologically advanced therapeutic strategies. Emerging approaches, including bioengineered biomaterials, self-responsive dressings, nanofiber scaffolds, cellular and molecular therapies and AI-assisted monitoring systems, are redefining wound management. These innovations move beyond passive coverage toward dynamic modulation of the wound microenvironment, targeted therapeutic delivery and personalized care. Collectively, the convergence of immunobiology, regenerative medicine, materials science and digital technologies signals a transition from symptomatic management to mechanism-driven regeneration. Continued interdisciplinary collaboration and translational rigor will be essential to translate these advances into safe, scalable and clinically effective solutions for patients with complex wounds.
